# Connecting Metainflammation and Neuroinflammation Through the PTN-MK-RPTPβ/ζ Axis: Relevance in Therapeutic Development

**DOI:** 10.3389/fphar.2019.00377

**Published:** 2019-04-12

**Authors:** Gonzalo Herradon, M. Pilar Ramos-Alvarez, Esther Gramage

**Affiliations:** ^1^Departamento de Ciencias Farmacéuticas y de la Salud, Facultad de Farmacia, Universidad San Pablo-CEU, CEU Universities, Madrid, Spain; ^2^Departmento de Química y Bioquímica, Facultad de Farmacia, Universidad San Pablo-CEU, CEU Universities, Madrid, Spain

**Keywords:** pleiotrophin, midkine, PTPRZ, neuroinflammation, inflammation, aging, obesity, neurodegeneration

## Abstract

Inflammation is a common factor of pathologies such as obesity, type 2 diabetes or neurodegenerative diseases. Chronic inflammation is considered part of the pathogenic mechanisms of different disorders associated with aging. Interestingly, peripheral inflammation and the associated metabolic alterations not only facilitate insulin resistance and diabetes but also neurodegenerative disorders. Therefore, the identification of novel pathways, common to the development of these diseases, which modulate the immune response and signaling is key. It will provide highly relevant information to advance our knowledge of the multifactorial process of aging, and to establish new biomarkers and/or therapeutic targets to counteract the underlying chronic inflammatory processes. One novel pathway that regulates peripheral and central immune responses is triggered by the cytokines pleiotrophin (PTN) and midkine (MK), which bind its receptor, Receptor Protein Tyrosine Phosphatase (RPTP) β/ζ, and inactivate its phosphatase activity. In this review, we compile a growing body of knowledge suggesting that PTN and MK modulate the immune response and/or inflammation in different pathologies characterized by peripheral inflammation associated with insulin resistance, such as aging, and in central disorders characterized by overt neuroinflammation, such as neurodegenerative diseases and endotoxemia. Evidence strongly suggests that regulation of the PTN and MK signaling pathways may provide new therapeutic opportunities particularly in those neurological disorders characterized by increased PTN and/or MK cerebral levels and neuroinflammation. Importantly, we discuss existing therapeutics, and others being developed, that modulate these signaling pathways, and their potential use in pathologies characterized by overt neuroinflammation.

## Neuroinflammation and CNS Disorders

Activation of the innate immunity in the Central Nervous System (CNS) is a critical step in the healing process after brain injury. However, cumulative evidence points to deleterious effects of chronic neuroinflammation (Szepesi et al., 2018). Activation of astrocytes and microglia is a hallmark of neuroinflammation. Activated glial cells release free radicals, cytokines and other pro-inflammatory factors. Activated microglia can be prophylactic and protect the brain from a wide variety of events including traumatic injury, stroke or neurodegenerative diseases, through modulation of neuronal synapses, by promoting neurogenesis, clearing debris, and suppressing inflammation (Chen and Trapp, 2016). However, exacerbated or persistent neuroinflammation can potentially exert negative effects on CNS integrity and function accompanying a wide variety of CNS diseases (Kielian, 2016). Sustained neuroinflammation is implicated in the progressive nature of neurodegenerative diseases (Faden et al., 2016; Gasiorowski et al., 2017). In addition to the peripheral stimuli that can cause chronic neuroinflammation, inflammatory processes in the CNS are also observed in response to different central events such as contact with neurotoxins or traumatic brain injury (TBI) (Spielman et al., 2014) and have been related to vascular injury and angiogenesis (Popp et al., 2017). The perpetuation of these inflammatory processes can subsequently cause acute secondary injury, leading to chronic neurodegenerative diseases (Simon et al., 2017). Supporting this role of neuroinflammation in neurodegenerative diseases, genome-wide association studies have shown associations with neurodegenerative disorders susceptibility, such as Parkinson’s disease (PD), in the human leukocyte antigen region and other polymorphisms related to the immune system including inflammatory cytokines and their receptors (Nalls et al., 2014). In addition, chronic neuroinflammation has been shown to contribute to neuronal loss by apoptosis in different pathologies, including multiple sclerosis (Mori F. et al., 2014; Mandolesi et al., 2017), drug addiction and alcoholism, and psychiatric disorders such as major depression (Montesinos et al., 2016; Neupane, 2016). In the light of this dual role of neuroinflammation and microglial activation in health and disease, the identification of the mechanisms responsible for maintenance of microglial activation and associated continuous neuron damage is critical to understand the etiology and pathology of diseases characterized by overt neuroinflammation.

### Pleiotrophin and Midkine: Novel Modulators of Neuroinflammation

**FIGURE 1:**
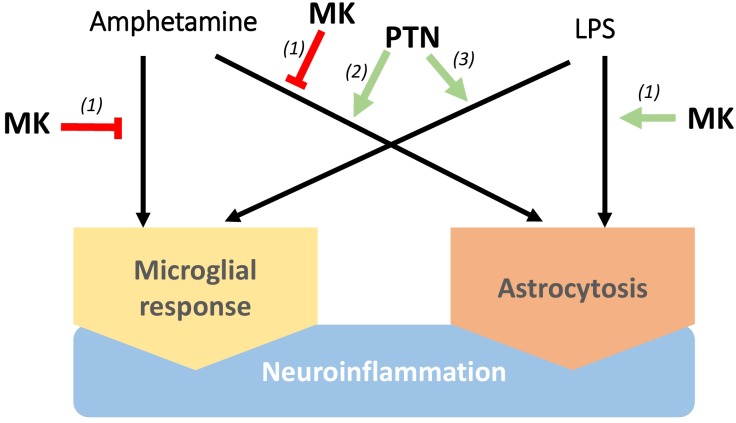
Differential regulation of neuroinflammation by Pleiotrophin (PTN) and Midkine (MK). MK lessens microglia responses and astrocytosis induced by amphetamine in the striatum, although lipopolysaccharide (LPS)-induced neuroinflammation seems to be potentiated by MK. On the other hand, PTN enhances neuroinflammation induced by different stimuli, including microglial activation, increase of pro-inflammatory cytokines in the brain and astrocytosis. (1) Vicente-Rodriguez et al. (2016a), (2) Vicente-Rodriguez et al. (2016b), (3) Fernandez-Calle et al. (2017).

In neuroinflammatory processes, the activation of innate immunity, contribution of Toll-like receptors (TLRs) and induction of expression of inflammation-related molecules such as cytokines, have been described in detail. Although the discovery of new modulators of neuroinflammation can potentially translate in novel targets and biomarkers for different diseases, the genetic bases of neuroinflammation are still far from being fully understood. Recently, we identified Pleiotrophin (PTN) and Midkine (MK) as two novel neurotrophic factors that modulate neuroinflammation in different contexts (Figure 1; Vicente-Rodriguez et al., 2016a,b; Fernandez-Calle et al., 2017). Pleiotrophin and MK are the only cytokines that constitute the *Ptn/Mk* developmental gene family (Kadomatsu et al., 1988; Milner et al., 1989). The pattern of expression of these cytokines in developing and adult nervous system has been extensively described in different species and led to study the importance of these cytokines in neural-glial interactions (Vanderwinden et al., 1992; Silos-Santiago et al., 1996; Xu et al., 2014). They are widely expressed during development but, in adults, their pattern of expression is restricted to a few cell types in different organs, including the brain, where they are mainly expressed in neurons. Importantly, the levels of expression of MK and PTN are highly upregulated after injury in different cells including microglia and inflammatory macrophages (Jin et al., 2009; Martin et al., 2011; Muramatsu, 2011; Gonzalez-Castillo et al., 2014). As suggested by their similar pattern of expression, PTN and MK overlap many functions including wound repair and survival of neurons (Gramage and Herradon, 2011; Herradon and Perez-Garcia, 2014). Accordingly, PTN and MK are upregulated in senile plaques and sera of patients with Alzheimer’s disease (AD) and in the substantia nigra of patients with PD among other neurodegenerative disorders (Herradon and Perez-Garcia, 2014). Midkine is also found in glial cytoplasmic inclusions of multiple system atrophy brains (Kato et al., 2000). In addition, both cytokines are also upregulated in the brain in pathological conditions characterized by neuroinflammation such as different types of brain injury, including ischemia, and after administration of drugs of abuse like amphetamine, alcohol and opioids (Table 1; Herradon and Perez-Garcia, 2014). It has been demonstrated that *Ptn*^-/-^ mice exhibit exacerbated amphetamine-induced dopaminergic injury in the nigrostriatal pathway (Gramage et al., 2010), which correlated with changes in the striatal phosphoproteome similar to those found in PD (Gramage et al., 2013). Other authors have demonstrated that *Ptn* overexpression in the brain exerts neurotrophic effects in rodent models of PD (Gombash et al., 2012). Taken together, these findings suggest that PTN protects against dopaminergic neurodegeneration in different pathological contexts.

**Table 1:** Pleiotrophin and Midkine expression is upregulated in different pathologies with a neuroinflammatory component.

Neuroinflammatory conditions	Pleiotrophin upregulation	Midkine upregulation
Amphetamine	Le Greves, 2005	–
Alcohol	Vicente-Rodriguez et al., 2014	Flatscher-Bader and Wilce, 2008; He et al., 2015
Opioid	Garcia-Perez et al., 2015	Ezquerra et al., 2007; Garcia-Perez et al., 2015
Neuropathic pain	Ezquerra et al., 2008	–
Parkinson’s disease	Marchionini et al., 2007	–
Alzheimer’s disease	Skillback et al., 2017	Yasuhara et al., 1993; Xiong et al., 2019
Brain injury	Yeh et al., 1998; Poulsen et al., 2000; Iseki et al., 2002	Yoshida et al., 1995; Mochizuki et al., 1998; Wada et al., 2002; Otsuka et al., 2016
Other neurodegenerative disorders	–	Yasuhara et al., 1996; Kato et al., 2000


*The upregulation of these cytokines in the CNS has been described after different stimuli or in pathologies related to neuroinflammatory processes.*

Midkine exerts relevant functions in peripheral inflammatory processes in different pathological conditions (Muramatsu, 2014; Weckbach et al., 2014), facilitating the migration of macrophages and neutrophils (Takada et al., 1997; Horiba et al., 2000; Sato et al., 2001) and preventing differentiation of regulatory T-cells (Wang et al., 2008; Sonobe et al., 2012). In the CNS, we found that amphetamine-induced microglial response and astrocytosis are enhanced in the striatum, but not in the hippocampus, of *Mk*^-/-^ mice. In contrast, lipopolysaccharide (LPS)-induced striatal astrogliosis was blocked by genetic inactivation of *Mk*, suggesting a differential regulation of astrocytosis by MK depending on the inflammatory stimulus (Figure 1; Vicente-Rodriguez et al., 2016a). On the other hand, *Ptn* overexpression in the brain (*Ptn*-Tg mice) potentiates the striatal astrocytosis induced by acute administrations of amphetamine (Vicente-Rodriguez et al., 2016b). Pleiotrophin overexpression also potentiates microglial activation after an acute systemic administration of LPS (Fernandez-Calle et al., 2017). Importantly, LPS-induced increases of pro-inflammatory cytokines in the brain, including TNF-α and IL-1β, were significantly enhanced in *Ptn*-Tg mice (Fernandez-Calle et al., 2017). The data suggest that PTN potentiates the acute neuroimmune response induced by different stimuli, including microglial activation (Figure 1), which is necessary and critical for host defense (Chen and Trapp, 2016) and could contribute to the known neurotrophic effects of this cytokine. However, although upregulation of PTN levels in the brain has been described in different chronic diseases such as AD and PD (Herradon and Perez-Garcia, 2014), in which long-term neuroinflammation is a pathological hallmark, the possible modulatory role of PTN in persistent neuroinflammation remains to be elucidated. If chronic upregulation of PTN levels in these diseases contributes to prolonged neuroinflammation, PTN would parallel the dual role of glial activation in health and disease depending on the duration of its actions. Although further studies are needed to better understand the roles of PTN and MK in the regulation of neuroinflammation in chronic CNS disorders, evidence strongly suggests that pharmacological modulation of the actions of PTN and MK is a novel strategy to treat disorders characterized by neuroinflammation.

It has to be noted that aging-associated neuroinflammatory conditions (e.g., AD) have been related with vascular injury and angiogenesis (Popp et al., 2017) and PTN expression levels are associated strongly with aging in human cerebrospinal fluid (Wyss-Coray, 2016). Both PTN and MK induce tumor growth mainly by promoting angiogenesis (Perez-Pinera et al., 2008; Weckbach et al., 2012; Papadimitriou et al., 2016). Pleiotrophin exerts mitogenic activity in fibroblasts (Milner et al., 1989), brain capillary endothelial cells (Courty et al., 1991), and in adrenal carcinoma cells (Fang et al., 1992). Importantly, *Ptn* is upregulated in vascular endothelial cells in sites of neovascularization after focal cerebral ischemia (Yeh et al., 1998). The angiogenic potential of PTN has been shown in studies in ischemic myocardium in rats, in which it was demonstrated that PTN stimulates significant increases in normal appearing new capillaries, arterioles, and newly formed blood vessels that interconnect with the existent coronary vascular system (Christman et al., 2005a). On the other hand, MK promotes endothelial cell proliferation and the recruitment of inflammatory cells to lesions (Kadomatsu et al., 2014). As in the case of PTN, an important pro-angiogenic role under hypoxia conditions has been described for MK (Weckbach et al., 2012). Thus, both cytokines have been proposed as important factors for inflammation, tissue repair and angiogenesis. Accordingly, it seems reasonable to propose the modulation of PTN/MK signaling pathways for the treatment of the above mentioned disorders characterized by neuroinflammation and vascular injury.

### The Mechanism of Action of Pleiotrophin and Midkine in Relation to Neuroinflammation

Midkine and PTN bind different receptors such as Receptor Protein Tyrosine Phosphatase (RPTP) β/ζ (Maeda et al., 1996, 1999; Meng et al., 2000), syndecan-3 (Rauvala et al., 2000), and anaplastic lymphoma kinase (ALK) (Stoica et al., 2001). Midkine also binds other proteoglycans (Muramatsu, 2011; Kadomatsu et al., 2013), including Neuroglycan C, through which MK has been shown to promote neurites in oligodendrocyte precursor-like cells (Ichihara-Tanaka et al., 2006). Also, MK has been shown to bind low density lipoprotein receptor-related protein (LRP), integrin α4β1 and integrin α6β1 in the brain (Muramatsu et al., 2000, 2004).

RPTPβ/ζ (a.k.a. RPTPβ, PTPRZ, and PTPζ) (Krueger and Saito, 1992), is abundantly expressed in the CNS as a chondroitin sulfate proteoglycan (Krueger and Saito, 1992; Maeda et al., 1994). Pleiotrophin and RPTPβ/ζ are highly expressed in human white matter oligodendroglial precursor cells (OPCs), and PTN-PTPRZ signaling promotes postnatal OPC differentiation during developmental myelination and remyelination after injury (Harroch et al., 2002; Sim et al., 2006; Kuboyama et al., 2015; Tanga et al., 2019). RPTPβ/ζ is composed of an N-terminal carbonic anhydrase-like domain, a fibronectin type III domain, a serine, glycine-rich domain that is thought to be chondroitin sulfate attachment region, a transmembrane segment, and two tyrosine phosphatase domains (Krueger and Saito, 1992). There are four splice variants of this molecule including a full-length (PTPζ-A), a short form (PTPζ-B), the secreted form (PTPζ-S or 6B4 proteoglycan/phosphacan), which corresponds to the extracellular region of PTPζ-A (Maeda et al., 1994; Maurel et al., 1994) and the PSI isoform, expressed only in neurons (Heck et al., 2005). Midkine binds mostly to the chondroitin sulfate portion of RPTPβ/ζ (Maeda et al., 1999) which has been proven to be essential for MK-induced neuron survival effects. The interaction of RPTPβ/ζ with PTN inactivates the intrinsic tyrosine phosphatase activity of RPTPβ/ζ, presumably by enforcing a conformational change in RPTPβ/ζ that denies substrates access to its active site in the D1 domain (Meng et al., 2000).

These signaling pathways triggered by PTN and MK through RPTPβ/ζ support important roles of these cytokines in neuroinflammation (Figure 1) since they modulate the tyrosine phosphorylation of substrates of RPTPβ/ζ that are known regulators of neuroinflammation such as TrkA (Shintani and Noda, 2008) and Fyn kinase (Pariser et al., 2005; Panicker et al., 2015). After LPS administration, Fyn is activated in microglia (Panicker et al., 2015). Activated Fyn phosphorylates PKCδ at Y311, contributing to an increase in PKCδ kinase activity and activation of the NFκB pathway (Panicker et al., 2015), suggesting that the PTN/RPTPβ/ζ is a major regulatory pathway of this pro-inflammatory signaling cascade. These signaling events are also observed in animal models of PD (Panicker et al., 2015).

The wide pattern of expression of *Ptn* in developing and adult tissues, including nervous system and peripheral organs, has been described in detail (Vanderwinden et al., 1992; Xu et al., 2014). The sites of expression of RPTPβ/ζ in peripheral organs are highly relevant for its potential role in inflammation. RPTPβ/ζ is expressed in the intestine, mononuclear cells, monocytes, macrophages (Zwicker et al., 2016), in hematopoietic stem cells (HSC) and B cells, in which RPTPβ/ζ promotes HSC maintenance and B cell survival (Sorrelle et al., 2017). RPTPβ/ζ expression is increased in inflammatory processes associated with kidney injury, in which interleukin 34 (IL-34) increases its phosphatase activity and promotes monocyte and macrophage infiltration in the kidney, contributing to chronic renal damage (Sanchez-Nino et al., 2016). As in the case of PTN and MK, these findings point to an important role of RPTPβ/ζ in the regulation of central and peripheral inflammatory processes. However, the relevance of the changes in the phosphatase activity of RPTPβ/ζ may differ in acute and chronic inflammatory processes and has not been studied before, which is due, in part, to the lack of proper ligands of this receptor. Recently, Fujikawa et al. (2016) identified SCB4380 as a potent inhibitor of RPTPβ/ζ in a rat allograft model of glioblastoma. Unfortunately, the low lipophilicity of this inhibitor, which requires liposome carriers for intracellular delivery *in vitro*, is a critical limitation for its use in studies in the CNS. To fill this gap, we recently developed BBB permeable inhibitors of RPTPβ/ζ, selecting the lead compound (MY10) after *in vitro* and *in vivo* validation (Fernandez-Calle et al., 2018; Pastor et al., 2018).

## Metainflammation and Neuroinflammation

Inflammatory responses in the brain in response to metabolic stress have been observed in both mice and humans, but little is known about the mechanisms that activate them (Thaler et al., 2012). For instance, low level inflammation in response to an excess of nutrients or energy is not necessarily a pathological process but reflects on a series of potentially harmful alterations in metabolic homeostasis, which has been called metainflammation (Gregor and Hotamisligil, 2011). Chronic overfeeding is associated with excess peripheral pro-inflammatory mediators that contribute to neuroinflammation, which subsequently exacerbates neurodegeneration (Spielman et al., 2014). Accordingly, epidemiological studies indicate that AD and PD risk positively correlate with pro-inflammatory conditions such as diabetes mellitus or metabolic syndrome (Spielman et al., 2014). Thus, the discovery of new pharmacological targets involved in the preservation of a healthy basal metabolism would potentially translate in novel therapeutic interventions not only in metabolic diseases but in neuroinflammatory CNS disorders. In this context, we will now summarize the evidence demonstrating that PTN is essential to preserve the appropriate basal metabolism.

### Pleiotrophin: A Novel Modulator of Metainflammation, Insulin Resistance, and Adipose Tissue Plasticity

Although *Ptn* is expressed in adults (Vanderwinden et al., 1992), its role in adipose tissue is not fully understood. *Ptn* expression is found in human adipose tissue, being higher in visceral white adipose tissue (WAT) than in subcutaneous WAT (Hoggard et al., 2012). In addition, the expression of *Ptn* in mice also depends on the type of visceral deposit, being about 30 times higher in the mesenteric than in the perigonadal fat. Pleiotrophin deletion is associated with a lipodystrophic phenotype (Sevillano et al., 2012), altered energy metabolism and insulin resistance (Sevillano et al., 2019). These results, together with previous reports from our group showing that PTN is increased in the visceral WAT of obese pregnant women, point to a novel role of PTN in metabolic homeostasis and metainflammation.

The association of insulin resistance with inflammation has been studied in depth during the last few decades (Shoelson et al., 2006; Olefsky and Glass, 2010; Hotamisligil, 2017), including the role of macrophages (Olefsky and Glass, 2010). It has been shown that inflammatory cytokines activate kinases that promote Ser phosphorylation of insulin receptor substrate 1 (pSerIRS1) (Bluher et al., 2009; Copps and White, 2012), which is considered a marker of insulin resistance in adipose tissue, not only in pathological (Schmitz-Peiffer and Whitehead, 2003) but also physiological situations, as shown previously (Sevillano et al., 2005, 2007; de Castro et al., 2011). An additional factor is the heterogeneity of macrophages; M1 are pro-inflammatory while M2 are anti-inflammatory and participate in homeostasis and tissue remodeling (Stienstra et al., 2008). Peroxisome proliferator-activated receptor gamma (PPAR-γ) is key in metainflammation, since it inhibits the inflammatory response in macrophages (Welch et al., 2003). Thus, activators of PPAR-γ, such as thiazolidinediones (TZD), improve the insulin response of adipose tissue by inhibiting the production of inflammatory mediators, including TNF-α (Sharma and Staels, 2007), and by increasing the levels of adiponectin (Combs et al., 2002), an anti-inflammatory adipokine that is downregulated in states of insulin resistance such as obesity or type 2 diabetes (T2D) (Statnick et al., 2000). In the same manner, treatment with TZDs reverses the polarization toward the pro-inflammatory phenotype M1 in diet-induced obesity models (Stienstra et al., 2008; Fujisaka et al., 2009). Precisely, we have proposed very recently that defective PPAR-γ activation may underlie insulin resistance of *Ptn* deficient mice by promoting an inflammatory condition that impairs lipid and glucose homeostasis (Sevillano et al., 2019).

The great plasticity of WAT enables this tissue to contract or expand in response to alterations in the energy balance. It has been proposed that WAT plasticity is a key factor in metabolic alterations such as obesity (Carobbio et al., 2017). Expansion of adipose tissue can be caused by hypertrophy, due to accumulation of triacylglycerides and by hyperplasia characterized by adipogenesis. Numerous molecules participate in this process, such as WNT and its downstream effectors, transcription factors, such as PPAR-γ (Spiegelman, 1998), or oxidative stress (Lowe et al., 2011). Interestingly, it has been suggested that PTN could inhibit the expression of PPAR-γ, which would block white adipocyte differentiation (Gu et al., 2007). The role of PTN in this context, however, remains unclear, since rPTN has been found to decrease Pparγ2 expression *in vitro*, but the same study shows *in vivo* that injection of a PTN-neutralizing antibody slightly decreased Pparγ2 in adipose tissue (Wong et al., 2016). Beige adipocytes can develop in WAT, a process known as browning, in response to certain stimuli, such as treatment with TZDs or cold exposure (Wang and Seale, 2016). WAT browning and brown adipose tissue (BAT) (Bartelt and Heeren, 2014; Lee et al., 2014) may represent important therapeutic targets because they facilitate the oxidation of excess lipids (Kajimura et al., 2015), thus preventing hyperlipidaemia and potentially also lipotoxicity. In this context, it has been recently demonstrated that PTN impairs brown adipocyte differentiation (Sevillano et al., 2019). In fact, the lipodystrophic phenotype of *Ptn*^-/-^ mice is related to an enhanced thermogenesis in BAT (Sevillano et al., 2019). The molecular events responsible for these newly discovered functions of PTN seems to involve the extracellular matrix (ECM).

The plasticity of WAT also requires vascular remodeling and ECM (Pellegrinelli et al., 2016). Obesity is usually accompanied by infiltration of macrophages in the tissue, and it has been proposed that the matrix metalloproteinases (MMPs) secreted by resident macrophages in WAT participate in the degradation of ECM proteins and promote adipocyte-induced secretion of MMPs (Mariman and Wang, 2010), thus supporting WAT remodeling. In this context, it is important the phosphatase ADAMTS1, an MMP that contributes to the degradation of highly expressed collagens in WAT, such as Col1α1 (Mori S. et al., 2014). It has been proposed that ADAMTS1 plays a modulatory role in ECM and adipogenesis by inhibiting the differentiation of mesenchymal cells to preadipocytes. In fact, in a diet-induced obesity mouse model, expression of Adamts1 is decreased in the perigonadal WAT, which is consequently expanded by an activated adipogenesis. Accordingly, an inverse correlation between Adamts1 expression and body mass index (BMI) has been found in humans (Chen et al., 2016). It is important to note that ADAMTS1 induces *Ptn* expression through the WNT/β-catenin signaling pathway, suggesting a role of this cytokine in the effects of MMPs on adipocyte differentiation. This is supported by studies in humans with diet-induced weight gain that show concomitant increases in the levels of expression of ADAMTS1, PTN and different mediators of WNT signaling (Wong et al., 2016). Interestingly, preliminary results from our group showed that, when brown adipocytes differentiate, Adamts1 expression is blunted, which correlates with a decreased expression of *Ptn* (Sevillano et al., 2019).

### The Metainflammation- Neuroinflammation Connection: Role of Pleiotrophin

**FIGURE 2:**
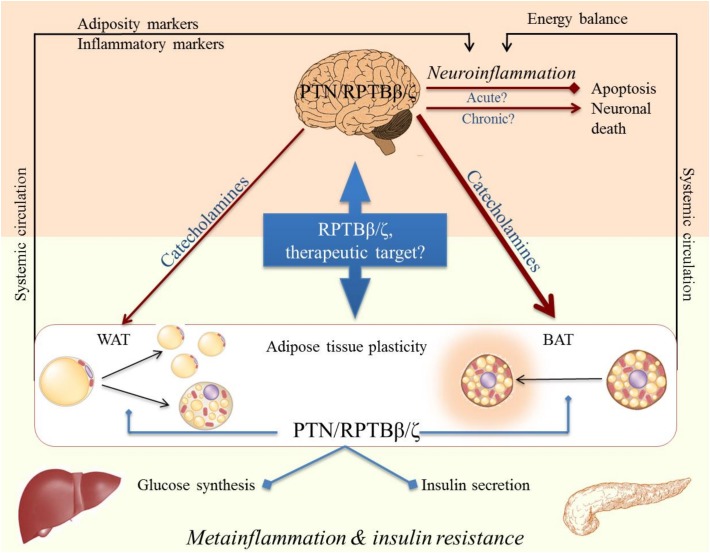
Cross-talk CNS-periphery in inflammation and its possible modulation by RPTBβ/ζ and its ligand Pleiotrophin (PTN).

Although metainflammation was first described in WAT, it may also affect other tissues such as the liver, pancreas, or hypothalamus. In fact, to maintain organism homeostasis, the brain must control the energy state of the periphery, as the CNS depends on the continuous supply of nutrients from the general circulation. Adipose tissue is innervated by the sympathetic endings of the autonomic nervous system, with BAT being much more innervated than WAT (Figure 2). It has been shown that CNS neurons are involved in the multisynaptic pathways to both liver and WAT, which allows a coordinated control of peripheral metabolism (Stanley et al., 2010). The alteration of any of the key components of this system, as it occurs in inflammation, or the failure of its integration could be involved in the etiopathogenesis of metabolic disorders such as obesity and diabetes, but also in CNS disorders such as neurodegenerative diseases (Figure 2; Santiago and Potashkin, 2014). As mentioned before, peripheral inflammatory diseases such as T2D and obesity confers an increased risk of developing AD and PD (Spielman et al., 2014), presumably through their capacity to cause prolonged low-grade neuroinflammation. It is conceivable, therefore, that bidirectional communication and cross-talk between the CNS and the periphery may be relevant to these pathological conditions (Figure 2). In fact, the CNS seems to be involved in the control of proliferation and differentiation of white adipocytes. Accordingly, *in vivo* surgical or pharmacological denervation of WAT triggers an increase in the number of white pre-adipocytes and adipocytes (Cousin et al., 1993; Bowers et al., 2004), whereas noradrenaline inhibits the proliferation of adipocyte precursor cells *in vitro* (Jones et al., 1992). On the other hand, when BBB homeostasis is altered, as it occurs in some metabolic disorders, the development of central pathological events such as neurodegeneration are facilitated (Mauro et al., 2014), probably by increasing brain sensitivity to peripheral stimuli that eventually end up causing chronic neuroinflammation. For instance, systemic administration of LPS is an animal model of peripherally-induced neuroinflammation and neurodegeneration (Qin et al., 2007, 2013), and there is evidence that peripheral infections accompanied by inflammation represent major risk factors for the development of sporadic AD and PD (Holmes, 2013; Su and Federoff, 2014). Other alterations of BBB such as pericyte dysfunction at the neurovascular unit have been linked with aberrant angiogenesis (Sweeney et al., 2016). As PTN is known to modulate the astrocytic response in different contexts and to play a role in vascular formation (Martin et al., 2013; Zhang et al., 2014; Wang et al., 2017; Fernandez-Calle et al., 2018), a dual function of this cytokine in both angiogenesis and neuroinflammation in these conditions should not be ruled out.

In addition, it has to be noted that PPAR-γ is known to be involved in degenerative processes in the brain and peripheral inflammation in pathologies such as DM or obesity (Villapol, 2018). Importantly, PPAR-γ is also associated with the control of angiogenesis in the brain (Arany et al., 2008). As explained before, PTN is involved in the regulation of PPAR-γ expression (Gu et al., 2007), suggesting the interesting possibility that this mechanism may underlie the common role of this cytokine in diabetes, neurodegeneration and angiogenesis.

It is also important to identify modulatory factors regarding the communication between the CNS and the immune system involved in the control of immune responses both centrally and peripherally. Extracellular vesicles (ExV), including exosomes, are released by all cells, including those of adipose tissue and nervous system. Their capacity to deliver a variety of bioactive molecules (e.g., protein, mRNA, miRNA, DNA, and lipids) to both nearby and distal cells suggests that exosomes and specific miRNAs play a role in cancer, diabetes, obesity, cardiovascular disease (Trajkovski et al., 2011) and CNS disorders, including neuroinflammation and PD (Leggio et al., 2017). In this context, it has to be noted that PTN is secreted by murine and human neural precursor cells (NPCs) (Qin et al., 2017), which is consistent with reports identifying PTN in the secretomes of various neural stem cell populations (Furuta et al., 2004; Lee et al., 2012).

Since adipocytes secrete large proportions of exosomes, it has been proposed that exosomes and miRNAs derived from fat play a role in pathologies related with insulin resistance (Thomou et al., 2017). Furthermore, there is evidence that exosomes can penetrate the BBB, moving from the peripheral circulation to the CNS (Matsumoto et al., 2017). It is also interesting to note that brain endothelial exosomes may be used as serological biomarkers for BBB status during neuroinflammation (Ramirez et al., 2018), pointing to a key role of exosomes in CNS-periphery cross-talk. The possible involvement of exosomes in PTN-mediated effects needs to be elucidated.

## Therapeutic Perspectives

Pleiotrophin and MK are novel modulators of neuroinflammation that play different roles depending on the inflammatory stimulus and the duration of the neuroinflammatory processes. Further studies are needed to fully understand the role of these cytokines on neuroinflammation, particularly in chronic neuroinflammation. However, in the light of evidence summarized here, it is reasonable to hypothesize that both potentiation and inhibition of the PTN/MK signaling pathways are novel therapeutic strategies to modulate neuroinflammation in acute and chronic pathological states. For instance, potentiation of the acute neuroinflammatory actions of these cytokines may exert therapeutic effects in different types of brain injury through limitation of the damage and improvement of brain repair. In terms of metainflammation, potentiation of PTN/MK signaling pathways may contribute to control the inflammatory condition associated to metabolic disorders. On the contrary, inhibiting the actions of these cytokines may be beneficial in chronic neuroinflammatory states that promotes exacerbation of neurodegeneration.

The administration of rMK and rPTN has been proposed in a number of CNS and peripheral injuries, rending already significant effects in preclinical models, particularly in the case of myocardial ischemia (Christman et al., 2005b; Fukui et al., 2008) and bone repair (Lamprou et al., 2014; Liedert et al., 2014). Gene therapy or stem cells overexpressing these genes have also been proposed as delivery systems of these cytokines in different animal models of peripheral and central disorders and have been reviewed elsewhere (Muramatsu, 2011; Herradon and Perez-Garcia, 2014; Kadomatsu et al., 2014).

The potential of therapeutic inhibition of MK and PTN has been studied in depth for the treatment of malignant diseases in different experimental models, including siRNA, shRNA, antibodies and RNA aptamers (Muramatsu, 2011; Kishida and Kadomatsu, 2014; Shi et al., 2017). Interestingly, an aptamer to MK has already been used to treat experimental autoimmune encephalitis (EAE) in mice (Wang et al., 2008), which is an animal model of multiple sclerosis.

Although promising, the above mentioned strategies to potentiate or inhibit the actions of PTN and MK in neuroinflammation in CNS disorders are commonly restricted by the limitation of the route of administration (e.g., intracranial). Thus, these effective therapeutic strategies in preclinical models of different diseases are not likely to progress through clinical development in the midterm. We will now summarize the existing modulators of PTN/MK signaling with approved therapeutic uses in humans and other promising strategies at the preclinical level that are more likely to progress through clinical development in the midterm.

### Potentiation of MK/PTN Actions

#### RPTPβ/ζ Inhibitors

Recently, Fujikawa et al. (2016) described SCB4380 as a potent inhibitor of PTPRZ (RPTPβ/ζ) as a potential new drug for glioma therapy. Unfortunately, its physicochemical properties make very difficult for this molecule to go through biological barriers. Thus, SCB4380 required liposome carriers for intracellular delivery (Fujikawa et al., 2016). To overcome this limitation in the RPTPβ/ζ inhibition strategy, novel BBB permeable selective small-molecule inhibitors of RPTPβ/ζ were recently designed and synthesized through rational drug design (Pastor et al., 2018). The lead compound, MY10, interacts with the intracellular domain PD1 of RPTPβ/ζ and inhibits its tyrosine phosphatase activity. MY10 has been validated *in vitro* and *in vivo*. Interestingly, systemic administration of MY10 reduces alcohol consumption and blocks the rewarding effects of alcohol in mice (Fernandez-Calle et al., 2018), replicating the effects obtained before with the transgenic mouse model (*Ptn-*Tg) overexpressing in the brain *Ptn*, the endogenous inhibitor of RPTPβ/ζ (Vicente-Rodriguez et al., 2014). Using these *Ptn-*Tg mice, it was demonstrated that PTN overexpression in the brain potentiates LPS-induced microglial activation and neuroinflammation (Fernandez-Calle et al., 2017), suggesting that acute treatment with MY10 would cause similar effects and could be used in brain repair after injury by promoting acute neuroimmune responses. However, persistent and/or over-activation of microglia is deleterious. Thus, more knowledge regarding the role of PTN in chronic neuroinflammation is needed to substantiate the pharmacological use of RPTPβ/ζ inhibitors as a potential therapeutic strategy in CNS disorders related with chronic neuroinflammation.

### Inhibition of MK/PTN Actions

#### ALK Inhibitors

ALK has been identified as a receptor for PTN (Stoica et al., 2001), but is also a substrate of RPTPβ/ζ (Perez-Pinera et al., 2007). It has been shown to mediate many of the central actions of PTN and MK (Herradon and Perez-Garcia, 2014; Dutton et al., 2017) and has been recently identified as a potent regulator of NLRP3 inflammasome activation in macrophages through its capacity to activate NF-kB (Zhang et al., 2018). Interestingly, it has been recently demonstrated that PTN activates AKT signaling through ALK to promote the morphological maturation and synaptic integration of newborn neurons (Tang et al., 2019). Pleiotrophin or MK-induced inhibition of RPTPβ/ζ causes an increase in tyrosine phosphorylation in ALK and subsequent activation of its tyrosine kinase activity (Perez-Pinera et al., 2007; Herradon et al., 2009).

Three generations of ALK inhibitors have been developed for the treatment of non–small cell lung cancer with certain mutations in Alk (Fan et al., 2018; Rothenstein and Chooback, 2018). Interestingly, these inhibitors are also effective in preclinical models of non-malignant completely unrelated pathological conditions. For instance, inhibition of ALK with alectinib, a second generation ALK inhibitor, significantly reduces alcohol binge drinking in rodents (Dutton et al., 2017). Inhibition of ALK with ceritinib and lorlatinib blocks NLRP3 inflammasome activation in macrophages (Zhang et al., 2018). Taking together, evidence suggests that the effects of the PTN-MK-RPTPβ/ζ axis on neuroinflammation could be modulated through pharmacologic regulation of one of its main effectors: ALK.

#### Fyn Inhibitors

Fyn kinase is a substrate of RPTPβ/ζ (Pariser et al., 2005). Fyn has been found activated in microglia in animal models of PD and after treatment with LPS and triggers the activation of the pro-inflammatory NFκB pathway in these models (Panicker et al., 2015). These data suggest that Fyn is a mediator of the actions of the PTN-MK-RPTPβ/ζ axis on neuroinflammation and, thus, pharmacologic inhibition of Fyn could be considered as a potential strategy to modulate the effects of this axis in diseases characterized by neuroinflammatory processes. In this context, it is interesting to note that Poli et al. (2018) have recently developed through rational drug design new derivatives of previous hit compounds Fyn inhibitors with considerable potency (IC_50_ = 0.76 μM).

#### MK Inhibitors

Small-molecule midkine inhibitors have been investigated mainly in the field of cancer therapy (Matsui et al., 2010). A promising compound (iMDK) has been recently proven to be effective in preclinical models of non–small cell lung cancer (Hao et al., 2013), oral squamous cell carcinoma (Masui et al., 2016) and prostate cancer (Erdogan et al., 2017). By inhibiting the actions of MK on RPTPβ/ζ, iMDK would leave unchecked the intrinsic phosphatase activity of this receptor. As a result, decreased phosphorylation and activation of its substrates (e.g., Fyn, ALK) would contribute to decreased neuroimmune responses.

## Conclusion

The expression of the components of the PTN-MK-RPTPβ/ζ axis in immune cells and in inflammatory diseases suggests important roles for this axis in inflammation. Pleiotrophin has been recently identified as a limiting factor of metainflammation, a chronic pathological state that contributes to neuroinflammation and neurodegeneration. Pleiotrophin also seems to potentiate acute neuroinflammation independently of the inflammatory stimulus while MK seems to play different -even opposite- roles in acute neuroinflammation depending on the stimulus. Which are the functions of MK and PTN in chronic neuroinflammation is still a question of great biologic interest.

For its pattern of expression and its known signaling cascades involving important regulators of inflammation as Fyn kinase and ALK, RPTPβ/ζ is a target receptor for PTN and MK in neuroinflammation. Pharmacologic modulation of the PTN, MK, RPTPβ/ζ and/or its downstream effectors, Fyn and ALK, is a novel therapeutic strategy to modulate neuroinflammation, from central or peripheral origin, in different pathological contexts.

## Author Contributions

GH and EG wrote the manuscript and built Figure 1 and Table 1. MPR-A wrote the manuscript and built Figure 2.

## Conflict of Interest Statement

The authors declare that the research was conducted in the absence of any commercial or financial relationships that could be construed as a potential conflict of interest.
